# HLA Allele and Haplotype Frequencies in Three Urban Mexican Populations: Genetic Diversity for the Approach of Genomic Medicine

**DOI:** 10.3390/diagnostics10010047

**Published:** 2020-01-16

**Authors:** Alma D. Del Angel-Pablo, Ana Itzel Juárez-Martín, Gloria Pérez-Rubio, Enrique Ambrocio-Ortiz, Luis A. López-Flores, Angel E. Camarena, Ramcés Falfán-Valencia

**Affiliations:** 1 HLA Laboratory, Instituto Nacional de Enfermedades Respiratorias Ismael Cosío Villegas, Mexico City 14080, Mexico; alyde_08@hotmail.com (A.D.D.A.-P.); glofos@yahoo.com.mx (G.P.-R.); acamarena@iner.gob.mx (A.E.C.); 2 Sección de Estudios de Posgrado e Investigación Escuela Superior de Medicina, Instituto Politécnico Nacional, Mexico City 11340, Mexico; 3 Centro de Estudios Antropológicos- Facultad de Ciencias Políticas y Sociales, Universidad Nacional Autónoma de México, Mexico City 04510, Mexico; ana.juarez@politicas.unam.mx

**Keywords:** HLA, Chihuahua, Tlalpan, Xalapa, haplotype, biobank, population genetics

## Abstract

Genetic variability defends us against pathogen-driven antigens; human leucocyte antigens (HLA) is the immunological system in charge of this work. The Mexican mestizo population arises mainly from the mixture of three founder populations; Amerindian, Spaniards, and a smaller proportion of the African population. We describe allele and haplotype frequencies of HLA class I (-*A* and *-B*) and class II (-*DRB1* and -*DQB1*), which were analyzed by PCR-SSP in Mexican mestizo from three urban populations of Mexico: Chihuahua-Chihuahua City (*n* = 88), Mexico City-Tlalpan (*n* = 330), and Veracruz-Xalapa (*n* = 84). The variability of the allele HLA class I and class II among the three regions of Mexico are in four alleles: HLA-A*24:02 (36.39%), -B*35:01 (16.04%), -DRB1*04:07 (17.33%), and -DQB1*03:02 (31.47%), these alleles have been previously described in some indigenous populations. We identified 5 haplotypes with a frequency >1%: HLA-A*02:01-B*35:01-DRB1*08:02-DQB1*04:02, A*68:01-B*39:01-DRB1*08:02-DQB1*04:02, A*02:01-B*35:01-DRB1*04:07-DQB1*03:02, A*68:01-B*39:01-DRB1*04:07-DQB1*03:02, and A*01:01-B*08:01-DRB1*03:01-DQB1*02:01. Also, the haplotype A*02:01-B*35:01-DRB1*08:02-DQB1*04:02 was identified in Tlalpan and Xalapa regions. Haplotype A*01:01-B*08:01-DRB1*03:01-DQB1*02:01 was found only in Tlalpan and Chihuahua. In the Xalapa region, the most frequent haplotype was A*24:02-B*35:01-DRB1*04:07-DQB1*03:02. These alleles and haplotypes have been described in Amerindian populations. Our data are consistent with previous studies and contribute to the analysis of the variability in the Mexican population.

## 1. Introduction

The human leukocyte antigen (HLA; known as MHC in other vertebrates) plays a central role in the recognition and presentation of antigens to the immune system and represents the most polymorphic gene cluster in the human genome [1]. This extensive polymorphism of the HLA genes among world populations results from selective pressures, including functional adaptations, particularly of bacteria, viruses, and parasites [2,3], which are particularly important in the understanding of human population variability. The HLA system in different populations is important in disease association, transplantation, and anthropological studies, among others. This genetic system located on the chromosomal region 6p21.3 encodes the HLA-class I (*HLA-A*, *-B*, and *-C*), class II (*HLA-DRA1, DRB* loci, *DQA1*, *-DQB1*, *-DPA1* and *-DPB1*), and class III (encoding mostly complement system proteins) [4]. The HLA class I and class II can be analyzed to compare populations and calculate genetic distances (e.g., correspondence analysis and dendrograms), which have become a feasible genetic marker between populations due to their correlation with the geographic spaces inhabited by human groups [5].

**Figure 1:**
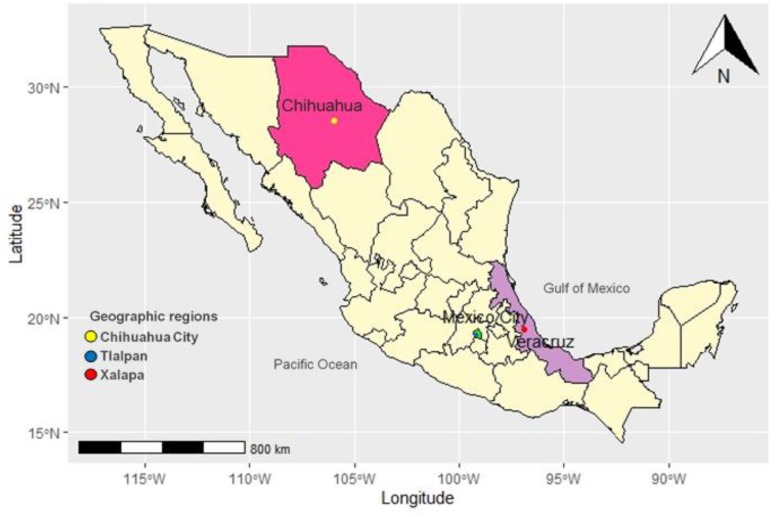
Geographical representation of Mexico showing the location of included populations. Chihuahua City (yellow point), located at the State of Chihuahua; Tlalpan (blue point), located at Mexico City, and Xalapa (red point), located at the State of Veracruz.

Mexico is located in the North American meridional region. The country’s territory has a total area of 1,972,550 km^2^ and a total population of 128,632,000 inhabitants; the national population comprises a high percentage of Spanish speakers (spa-ISO 639-3 Ethnologue); in addition, there are 70 indigenous groups speaking 69 native languages distributed throughout the territory [6,7] (Figure 1).

The genetic constitution of the Mexican population is very complex; at the time of the arrival of the Spanish conquistadors, the highest population density was located in the Mesoamerican region. After the Spanish conquest, indigenous groups settled in occupying the northern portion of the country came into contact with the Spaniards, who were attracted to this territory due to the discovery of large deposits of mineral resources along the road known as Real de Minas [8]. The present-day populations of northern Mexico are the result of a process of miscegenation between native Amerindian, Spanish, and African populations; the resulting genetic recombination of these populations allowed the emergence of new mestizo populations [9].

Mexico City is located in the central region of Mexico. It is the most highly populated city in the country, with 8,985,339 inhabitants [10]. Mexico City has been inhabited by several indigenous groups; indeed, the Nahua group is currently the largest in the region; mainly in the mayoralty of Milpa Alta [11,12,13]. Also, Mexico City is the economic, political, and social center of Mexico; most of its population speaks Spanish; nevertheless, some villages that are part of the city include Nahuatl speakers (nhw-ISO 639-3 Ethnologue) [7,11]. Tlalpan may have been the first urban settlement in the Basin of Mexico and was known as San Agustín de las Cuevas during the colonial period [14]. Tlalpan has a total population of 650,567 inhabitants, which includes both urban (29%) and rural areas [10] (Figure 1).

In the north of the country, the state of Chihuahua is bordered to the south by the states of Durango and Sinaloa, to the East by Coahuila, and to the west by Sonora. Chihuahua City, which is the capital and the second most important urban center of the state, has a population of 819,543 inhabitants [15] (Figure 1). Chihuahua City was founded in the XVIIth century as a mining center and Spanish military enclave. [16] Most of the population speaks Spanish; however, it includes a lower proportion of Tarahumara speakers (tar-ISO 639-3 Ethnologue) [7,12,17]. Currently, the majority of the population is dedicated to industrial, commercial, and tourist activities, as most of the inhabitants reside in urban areas [11,15,17].

The state of Veracruz is located in southern Mexico and is bordered to the north by the state of Tamaulipas, to the west by Hidalgo, Puebla, and Oaxaca, and to the southeast by the states of Tabasco and Chiapas. The total population of Veracruz is 8,127,832 inhabitants [18]. During the pre-Hispanic period, Veracruz was home to four important indigenous cultures: Huastec, Otomi, Totonacs, and Olmecs. Xalapa is the state capital of Veracruz and occupies 0.17% of the territorial surface of the state, which almost 50% consists of urban areas [19]. Numerous Spanish families established there, thus increasing the population, which is mostly composed of Spaniards and mestizos [11,12,17]. Currently, Xalapa is the second-most populous municipality of the state, with a population of 457,928 inhabitants [18], as shown in Figure 1.

The aim of this study was to report the allele and haplotype distribution of the HLA class I (*-A* and *-B*) and class II (-*DRB1* and *-DQB1*) genes among the Mexican population of three urban regions: Mexico City-Tlalpan, Chihuahua-Chihuahua City and Veracruz-Xalapa.

## 2. Materials and Methods 

### 2.1. The Sample

The participants were collected as controls for disease association studies. The study protocol was approved (approbation codes: B20-08, B05-10 and B20-15) by the Institutional Committee for Science and Ethics of the Instituto Nacional de Enfermedades Respiratorias Ismael Cosío Villegas (INER). After having been informed of the purpose of the research, all the volunteers signed a letter of consent and were provided an assurance-of-personal-data document.

A total of 502 subjects, all of whom were Mexican mestizos (MM) participants, were collected from three urban populations, then were divided into three geographic regions of Mexico: Chihuahua-Chihuahua City (Chihuahua, *n* = 88), Mexico City-Tlalpan (Tlalpan, *n* = 330), and Veracruz-Xalapa (Xalapa, *n* = 84). All the allele and haplotype frequencies can be checked at The Allele Frequency Net Database site with the following identification numbers: Mexico Mexico City Tlalpan (AFND-ID: 3655), Mexico Chihuahua Chihuahua City Pop 2 (AFND-ID: 3654) and Mexico Veracruz Xalapa (AFND-ID: 3653) (www.allelefrequencies.net).

### 2.2. HLA Typing

Genomic DNA was extracted from peripheral blood using the BDTrack DNA isolation kit (Maxim Biotech, San Francisco, CA, USA). Genotyping for HLA class I (*-A* and *-B*) and class II (*-DRB1* and *-DQB1*) was performed using PCR by Sequence-Specific Primers (PCR-SSP, (One Lambda Micro SSP™, Hannover Germany). Nomenclature for HLA genes was according to official WHO Nomenclature [20]. The two-fields resolution was performed by two steps. Firstly, typing was performed using a low-resolution technique (One Lambda Micro SSP™ Generic Trays, Hannover Germany), based on IMGT/HLA 3.23.0, which included *HLA-A*, *-B*, *-DRB1* and *-DQB1* specificities which can be from 8 to 48 independent well reactions, depending on the locus. Then, the allele discrimination of two fields and ambiguities resolution were done employing sets of high-resolution primers (One Lambda Micro SSP™ High-Resolution Trays, Hannover Germany), based on IMGT/HLA 3.23.0 with independent panels, oscillating from 22 to 48 primer-pairs, depending on the variability of each locus.

PCR-SSP methodology is based on the principle that oligonucleotide primers are used efficiently to amplify a target sequence [21]. The total number of primers used must amplify all known alleles (positive result), the PCR-SSP employed were low and high resolution, in order to solve the ambiguities generated by low resolution genotyping. This technique requires a pair of internal control primers (β-globin gene) for the entire amplification process, which serves to verify PCR reaction integrity. The pairs of primers were designed to have a perfect match with only one allele or group of alleles. In each well of the plate, we added DNA (150 ng/uL) to dried primers. Next, we added recombinant Taq polymerase (Thermo Scientific, Wilmington, DE, USA) and dNTP buffer mixture (Micro SSP D-mix). The amplification was carried out with Verity 96-Well thermal cycler (Applied Biosystems/Thermo Fisher Scientific Inc., Singapore) with a standardized amplification program [22]. After the PCR process, electrophoresis was performed to amplify DNA fragments on a 2.0% agarose gel and visualized by staining with 1.0% Ethidium Bromide (Sigma-Aldrich, St. Louis, MO, USA) with exposure to UV light transilluminator (UVP Inc. Upland, CA, USA) [23]. Finally, the interpretation of the PCR-SSP results was based on the presence or absence of a specific amplified DNA fragment that, using HLA Fusion™ 3.0 Software ((One Lambda, Inc. Canoga Park, CA, USA), identifies the alleles.

### 2.3. Statical Analysis and Data Visualization

Allele and haplotype frequencies were determined by Maximum-likelihood estimation (MLE) [24] using the software Arlequin v. 3.1 ( L. Excoffier, CMPG University of Berne, Berne, Switzerland) [25] and the Expectation-Maximization (EM) algorithm function in the total population and in the three regions. The observed versus expected heterozygosity (for each locus) was analyzed to determine the Hardy-Weinberg equilibrium (HWE). The linkage disequilibrium (LD) coefficient standardized D′ (∆′) was calculated according to Lewontin (1964) [26]. Absolute D′ values of 1 indicate complete LD; 0 corresponds to no LD [27]. Frequencies were compared using χ2 analysis in 2 × 2 contingency tables, as well as with Fisher’s exact test when appropriate; we consider *p* values that were ≤0.05 as statistically significant. The analysis was performed through Epi-Info v.7.2.2.6. R Studio v. 3.6.1 (R Core Team, Vienna, Austria) was used to create the Venn diagram and geographical map.

## 3. Results

In the whole-population analysis (Mexican mestizo, MM, *n* = 502), we identified 26 alleles for *HLA-A*, 54 alleles in *HLA-B*, 46 alleles in *HLA-DRB1,* and 16 in *HLA-DQB1*. We observed the highest diversity of alleles in the *-B* and *-DRB1* loci in our study population.

**Figure 2:**
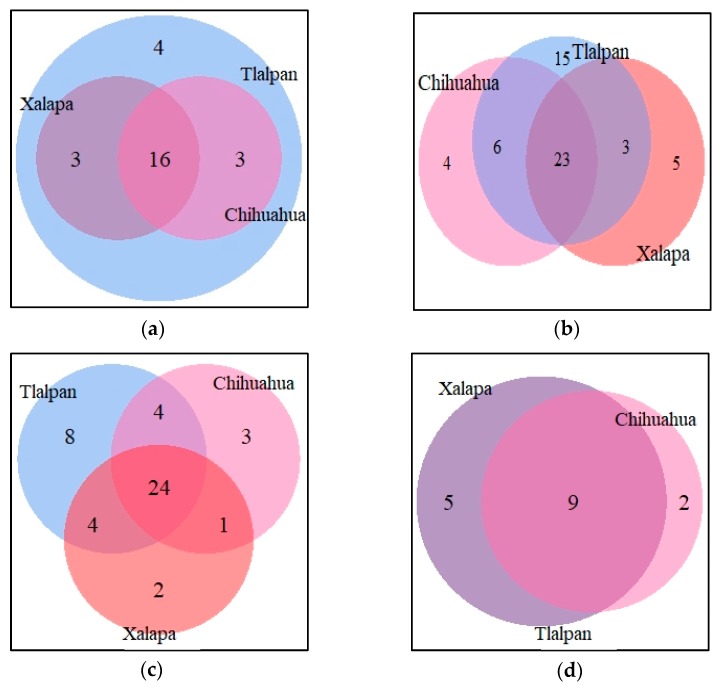
Venn diagram for the regions: Tlalpan, Chihuahua, and Xalapa. (**a**) *HLA-A* alleles shared in the three regions; (**b**) *HLA-B* alleles shared in the three regions, (**c**) *HLA-DRB1* alleles shared in the three regions, (**d**) *HLA-DQB1* alleles shared in the three regions.

The number of alleles in each locus in the three populations, and their distribution in the studied population, are shown in Figure 1. For the *HLA-A* locus, there are 16 alleles shared, while *HLA-B* shows 23 alleles; in Class II, *HLA-DRB1* has 24 alleles in common; and finally, in the *HLA-DQB1* locus only, alleles are in the three regions (Figure 2).

The Class I alleles (-*A* and *-B*) and Class II (-*DRB1* and -*DQB1*) data obtained for the three Mexican urban populations are provided in the supplementary information S1–S4. 

The estimates of HWE shows a deviation from expected/observed heterozygosity in MM for Class I loci: *HLA-A* (Obs. Het. = 0.8725/Exp. Het. = 0.8714; *p* = 0.013), and *HLA-B* (Obs. Het. = 0.9383/Exp. Het. = 0.9326; *p* = 0.007), but not for the Class II loci: *HLA-DRB1* (Obs. Het. = 0.9143/Exp. Het. = 0.9329; *p* = 0.104) and *HLA-DQB1* (Obs. Het. = 0.8327/Exp. Het. = 0.8186; *p* = 0.676). In the analysis of independent populations, the *HLA-A* and *-B* loci for the region Tlalpan differ in HWE (*p* < 0.05); while, for the regions Chihuahua City and Xalapa, the four loci did not differ significantly in HWE; the results from each region are shown in Table 1.

### 3.1. Allele Frequency

#### 3.1.1. HLA-A

In locus *HLA-A* for Tlalpan, the greatest variability was concentrated in 26 alleles; for Xalapa and Chihuahua, 19 alleles were found in each population. Alleles HLA-A*02:01, A*24:02, and A*68:01 were the most frequent in the Tlalpan and Xalapa region, while A*02:01, A*24:02, and A*01:01 in the Chihuahua region had the highest frequencies. We found statistically significant differences for the A*01:01 allele (*p* = 0.03) when comparing Tlalpan (6.06%) vs. Xalapa (1.79%). In the comparison of Tlalpan (13.79%) vs. Chihuahua (4.55%), statistically significant differences in the A*68:01 allele frequency (*p* = 0.001) were found, as were in the comparison Chihuahua (4.55%) vs. Xalapa (17.86%) (*p* < 0.001); in addition, differences in the allele A*01:01 (*p* < 0.004) were found. Table 2 shows the high-resolution for the *HLA-A* alleles with an allele frequency (AF) ≥ 1.0%. A full-length table depicting all alleles identified is available in Supplementary Table S1.

#### 3.1.2. HLA-B

For the *HLA-B* locus in the Tlalpan mayoralty, we reported 47 alleles, while for Veracruz-Xalapa we reported 31 alleles, and 33 alleles for the Chihuahua region. We found that HLA-B*35:01, B*39:01, and B*40:02 were present in Tlalpan at ~36%; in the Chihuahua region B*51:01, B*39:01, and B*35:01 alleles were found in a great part of the population, with a total frequency of 34%. In the Xalapa municipality, the alleles with higher frequency were similar to those described previously in Tlalpan, but the top-three almost reach 45%. According to the frequencies shown in Table 3, in the comparison of Tlalpan vs. Chihuahua regions, we found statistically significant differences in: B*07:02 (*p* = 0.034), B*14:01 (*p* = 0.027), B*48:01 (*p* = 0.027), and B*51:01 (*p* = 0.004). In Tlalpan vs. Xalapa regions, no statistically significant differences were found, and finally, in the Chihuahua vs. Xalapa comparison, statistically significant differences were detected in B*08:01 (*p* = 0.02), B*35:01 (*p* = 0.032), B*40:02 (*p* = 0.043), and B*51:01 (*p* < 0.03). Only alleles with AF ≥ 1.0% are included in Table 3. A full-length table depicting the frequencies of all alleles identified is available in Supplementary Table S2.

#### 3.1.3. HLA-DRB1

For the *HLA-DRB1* locus in the Tlalpan region, we found the greatest variability with a total of 42 alleles, compared to Chihuahua and Xalapa, with 32 and 30 alleles, respectively. The HLA-DRB1*04:07, DRB1*08:02, and DRB1*07:01 alleles were the most frequent in Tlalpan, in 36% of the population, while in Xalapa, the most frequent alleles were DRB1*04:07, DRB1*08:02, and DRB1*04:04, in ~45% of the population, and in Chihuahua, DRB1*04:04, DRB1*04:07, DRB1*07:01, and DRB1*08:02 were found in 34% of the population. When comparisons were performed, interestingly, we found statistically significant differences in the allele DRB1*04:07 for the Tlalpan (16.67%) vs. Chihuahua (10.23%) comparison (*p* < 0.05), Tlalpan (16.67%) vs. Xalapa (27.38%) comparison (*p* = 0.002), and Chihuahua (10.23%) vs. Xalapa (27.38%) comparison (*p* < 0.001). When Tlalpan vs. Chihuahua regions were compared, we found statistically significant differences in the allele DRB1*14:01 (*p* < 0.001); and in Tlalpan vs. Xalapa regions, for the alleles DRB1*03:01 (*p* = 0.013), DRB1*04:11 (*p*= 0.025), and DRB1*14:06 (*p* = 0.042). Finally, in Chihuahua vs. Xalapa: DRB1*03:01 (*p* < 0.02). Only alleles with AF ≥ 1.0% are included in Table 4. A full-length table depicting the frequencies of all alleles identified is available in Supplementary Table S3.

#### 3.1.4. HLA-DQB1

For *HLA-DQB1* locus, the alleles DQB1*03:01, DQB1*03:02, and DQB1*04:02 were found in higher frequency in Tlalpan (65%) and Xalapa (75.9%). For the population of Chihuahua, the alleles with higher occurrence were DQB1*03:02, DQB1*03:01, and DQB1*05:01 with 59.1% of the whole population. Interestingly, we found only 11 alleles in the Chihuahua region, compared with 14 alleles in the other two regions. When comparisons were made, statistically significant differences were obtained when we compared Tlalpan (2.42%) vs. Chihuahua (5.68%) (*p* < 0.05) for the allele DQB1*06:03. When Tlalpan vs. Chihuahua regions were compared, we found statistically significant differences in the alleles DQB1*02:01 (*p* = 0.009), DQB1*02:02 (*p* = 0.043), and DQB1*03:02 (*p* = 0.001). In Tlalpan vs. Chihuahua: DQB1*02:02 (*p* < 0.004), DQB1*03:02 (*p* < 0.001), and DQB1*05:01 (*p* = 0.028). The AF for those alleles > 1% is observed in Table 5. A full-length table depicting the frequencies of all alleles identified is available in Supplementary Table S4.

### 3.2. Haplotype Frequency

In the whole population, 761 haplotypes were detected, shared by the four loci. The Tlalpan population was where most haplotypes were identified (514), followed by Chihuahua (166), and Xalapa (149). Similarly, we reported five haplotypes with a frequency >1.0% in the MM population: HLA-A*02:01-B*35:01-DRB1*08:02-DQB1*04:02, A*68:01-B*39:01-DRB1*08:02-DQB1*04:02, A*02:01-B*35:01-DRB1*04:07-DQB1*03:02, and A*68:01-B*39:01-DRB1*04:07-DQB1*03:02. Table 6 shows the haplotype frequencies for those that have a haplotype frequency (HF) >1.0%.

In the Tlalpan region, the most frequent haplotypes were: HLA-A*02:01-B*35:01-DRB1*08:02-DQB1*04:02 (HF = 1.97%), A*68:01-B*39:01-DRB1*08:02-DQB1*04:02 (HF = 1.97%), A*02:01-B*35:01-DRB1*04:07-DQB1*03:02 (HF = 1.52%), A*68:01-B*39:01-DRB1*04:07-DQB1*03:02 (HF = 1.06%), and A*01:01-B*08:01-DRB1*03:01-DQB1*02:01 (HF = 1.06%). A table including these haplotypes with an HF >1% is available in Supplementary Table S5.

In Chihuahua, we found haplotypes with frequencies higher than 1.0% and the most frequent were HLA-A*01:01-B*08:01-DRB1*03:01-DQB1*02:01 (HF = 2.27%), followed by A*24:02-B*39:01-DRB1*04:07-DQB1*03:02 (HF = 1.70%). A table including haplotypes with an HF >1.0% is available in Supplementary Table S5.

For Xalapa, we reported 11 haplotypes with a frequency >1.0%; the haplotype with the highest frequency (2.98%) was HLA-A*24:02-B*35:01-DRB1*04:07-DQB1*03:02, followed by A*02:01-B*35:01-DRB1*04:07-DQB1*03:02, and A*02:01-B*35:01-DRB1*08:02-DQB1*04:02; both had a frequency of 2.38%. A table including haplotypes with an HF >1.0% is available in Supplementary Table S5.

## 4. Discussion

Mexico’s population is mostly composed of Mestizos, as with other Latin American populations, which are a recently admixed population composed of Amerindian, European and, to a lesser extent, African and Asian ancestries. In this matter, an important role of ethnicity in the susceptibility to different inflammatory/autoimmune and infectious diseases has been attributable to the inclusion of HLA alleles by miscegenation with Caucasian, Asian, and African populations. Nevertheless, studies of the genetics of diseases are difficult to replicate due to the complex nature of the environmental factors and the degree of genetic variability among human populations.

Comparative analyses between Mexicans and other neighboring populations reveal significant differences in genetic diversity [28]. The HLA allele-distribution varies between distinct populations; in our study the alleles HLA-A*02:01, B*35:01, DRB1*04:07, and DQB1*03:02 were found at the highest frequencies in the three studied regions. These alleles have been described in various Amerindian (Native Americans) groups, such as the Nahuas [29]. In the Tarahumara indigenous population, HLA-A*24:02:01, B*40:02, DRB1*08:02:01, DQB*04:02 were described with a frequency greater than 10% [30], and these alleles have also been found in our study at a frequency > 5% in each region; interestingly, the HLA-B*40 has been reported in other Amerindian populations [31]. For HLA class II, DRB1*04:07 and DQB1*03:02 alleles have been reported mainly in the Amerindian population; in Mexico, it has been reported more frequently in the Mayos population to the northeast of the country [32], and these alleles have been found to be the most frequent for the three regions analyzed in our study.

Similarly, the five haplotypes that showed the highest frequency in Tlalpan mayoralty were HLA-A*02:01-B*35:01-DRB1*08:02-DQB1*04:02, A*68:01-B*39:01-DRB1*04:07-DQB1*03:02, A*68:01-B*39:01-DRB1*08:02-DQB1*04:02, and A*02:01-B*35:01-DRB1*04:07-DQB1*03:02, which have been reported as Amerindian haplotypes of indigenous groups such as the Teneek, Seri, and Mayos [33], while the haplotype A*01:01-B*08:01-DRB1*03:01-DQB1*02:01 was principally reported in Caucasian population [34].

In Mexico’s Chihuahua, the most frequent haplotype was HLA-A*01:01-B*08:01-DRB1*03:01-DQB1*02:01. This haplotype has been reported in the European population, while A*24:02-B*39:01-DRB1*04:07-DQB1*03:02 has been described in ethnic groups such as the Mayos and Mazatecas [35].

For the Xalapa population, the haplotype with the highest frequency was A*24:02-B*35:01-DRB1*04:07-DQB1*03:02, followed by A*02:01-B*35:01-DRB1*04:07-DQB1*03:02 and A*02:01-B*35:01-DRB1*08:02-DQB1*04:02. Other studies have described these haplotypes as frequent in native Amerindians [33,36]. These results showed that most of the haplotypes found in the Tlalpan and Xalapa population were of Amerindian origin, while one haplotype of Caucasian origin was found at a higher frequency in Chihuahua compared with the other two populations, probably as a product of contact with the Spaniards conquers, who were attracted by the discovery of large deposits of mineral resources [8].

In some populations, genetic associations have been described with the presence of HLA alleles with different clinical phenotypes/diseases. The HLA-B*35 has been associated with increased risk of developing pulmonary arterial hypertension in systemic sclerosis patients [37], and we found a high frequency of this allele in our Mexican mestizo population; remarkably, it has been reported at a greater proportion in the Xalapa region compared to Tlalpan and Chihuahua. In our study population, we found the HLA class II alleles, DRB1*04:04 and DRB1*04:05, which have been described as associated with susceptibility to rheumatoid arthritis [38,39,40], while the DRB1*03:01 allele with Systemic Lupus Erythematosus [41,42]. Another allele associated with autoimmune diseases is HLA-A*01:01, which has been associated with the development of psoriatic arthritis in the Chinese population [43]. In a pilot study in the Mexican population, HLA-DQB1*05 was associated with susceptibility of reinfection with human papillomavirus [44]. Two alleles (HLA-DRB1*03:01 and DRB1*04:04) that we found in greater proportions in our study have been conferred with an increased risk of Addison’s disease [45]. Diabetes mellitus type 1 is one of the most complex diseases with the highest incidence in Mexico; the DQB1*05:01 and DQB1*02:01 alleles that occur in linkage disequilibrium with the DRB1*03:01 [46] allele, are presented with a frequency >1%. Interestingly, the DRB1*03: 01-DQB1* 02:01 haplotype was found as the most frequent haplotype in the Chihuahua region.

It is important to know the relationship between HLA alleles and the development of diseases, as some alleles are distributed in higher proportions in different regions of Mexico, which may act as a form of epidemiological monitoring. Our results are important for future comparative genetic studies in different Latin American ethnic groups, particularly Mexican Mestizos and Amerindians.

## 5. Conclusions

The alleles HLA-A*02:01, -B*35:01, -DRB1*04:07, and -DQB1*03:02, as well as the A*02:01-B*35:01-DRB1*08:02-DQB1*04:02 and A*68:01-B*39:01-DRB1*04:07-DQB1*03:02 haplotypes were found with higher frequencies in the whole Mexican mestizo population studied. Our results show the existence of alleles and haplotypes that have been reported with an increased frequency in Amerindian populations as well as others from Caucasian populations. The current data contribute to the understanding of genetic diversity in Mexico and serve to extend our knowledge of genetic variants of critical relevance for the development of genomic medicine in Mexico.

## Figures and Tables

**Table 1:** Hardy-Weinberg equilibrium (HWE) parameters for the sample sets from three Mexican populations.

Population		HLA-A	HLA-B	HLA-DRB1	HLA-DQB1
**MM**	Obs. Het.	0.8725	0.9383	0.9143	0.8327
	Exp. Het.	0.8714	0.9326	0.9329	0.8186
	*p*-value	0.0127 *	0.0069 *	0.1043	0.6764
**Tlalpan**	Obs. Het.	0.8636	0.9364	0.9273	0.8546
	Exp. Het.	0.8712	0.9328	0.9324	0.8214
	*p*-value	0.0411 *	0.0221 *	0.1350	0.4558
**Chihuahua**	Obs. Het.	0.9091	0.9546	0.9205	0.8296
	Exp. Het.	0.8753	0.9371	0.9494	0.8510
	*p*-value	0.1074	0.0729	0.4980	0.5634
**Xalapa**	Obs. Het.	0.8691	0.9286	0.8571	0.7500
	Exp. Het.	0.8602	0.9146	0.8954	0.7457
	*p*-value	0.9130	0.3824	0.0939	0.6401

MM: Mexican mestizo, whole population. Obs. Het.: Observed heterozygosity. Exp. Het.: Expected heterozygosity under HWE conditions. The *p*-value refers to the difference between the observed and expected heterozygosity values. * Only *p*-values < 0.05 are considered statistically significant.

**Table 2:** *HLA-A* allele frequency from Mexico: Tlalpan, Chihuahua, and Xalapa.

Allele	MM		Tlalpan		Chihuahua		Xalapa		*p*-Value		
									Tlalpan vs.		Chihuahua vs. Xalapa
	*n*	AF	*n*	AF	*n*	AF	*n*	AF	Chihuahua	Xalapa	
A*01:01	59	5.88	40	6.06	16	9.09	3	1.79		0.0300	0.0036
A*02:01	265	26.39	171	25.91	48	27.27	46	27.38			
A*02:02	20	1.99	16	2.42	1	0.57	3	1.79			
A*03:01	48	4.78	29	4.39	10	5.68	9	5.36			
A*11:01	27	2.69	18	2.73	6	3.41	3	1.79			
A*23:01	30	2.99	18	2.73	6	3.41	6	3.57			
A*24:02	165	16.43	113	17.12	28	15.91	24	14.29			
A*26:01	33	3.29	19	2.88	9	5.11	5	2.98			
A*29:01	29	2.89	18	2.73	8	4.55	3	1.79			
A*30:01	34	3.39	26	3.94	4	2.27	4	2.38			
A*31:01	65	6.47	34	5.15	15	8.52	16	9.52			
A*32:01	17	1.69	9	1.36	4	2.27	4	2.38			
A*33:01	19	1.89	13	1.97	3	1.7	3	1.79			
A*36	18	1.79	10	1.52	4	2.27	4	2.38			
A*68:01	129	12.85	91	13.79	8	4.55	30	17.86	0.0011		0.0002
A*68:03	10	1.00	9	1.36	0	0.00	1	0.60			
**Others**	36	3.58	26	3.94	6	3.42	4	2.35			

AF: Allele Frequency. Only alleles with AF ≥ 1.0% are included in this table. *n*: Refers to the number of alleles. Only *p*-values < 0.05 are considered significant.

**Table 3:** *HLA-B* allele frequency from Mexico: Tlalpan, Chihuahua, and Xalapa.

Allele	MM		Tlalpan		Chihuahua		Xalapa		*p*-Value		
									Tlalpan vs.		Chihuahua vs. Xalapa
	*n*	AF	*n*	AF	*n*	AF	*n*	AF	Chihuahua	Xalapa	
B*07:02	47	4.68	23	3.48	16	9.09	8	4.76	0.0336		
B*08:01	30	2.99	20	3.03	9	5.11	1	0.60			0.0200
B*13:01	12	1.20	9	1.36	2	1.14	1	0.60			
B*14:01	24	2.39	12	1.82	9	5.11	3	1.79	0.0270		
B*14:02	25	2.49	22	3.33	3	1.70	0	0.00			
B*15:01	54	5.38	36	5.45	7	3.98	11	6.55			
B*15:02	10	1.00	10	1.52	0	0.00	0	0.00			
B*18:01	25	2.49	15	2.27	7	3.98	3	1.79			
B*35:01	161	16.04	109	16.52	19	10.80	33	19.64			0.0324
B*35:02	43	4.28	28	4.24	5	2.84	10	5.95			
B*38:01	11	1.10	3	0.45	6	3.41	2	1.19			
B*39:01	131	13.05	86	13.03	20	11.36	25	14.88			
B*39:02	23	2.29	21	3.18	1	0.57	1	0.60			
B*39:06	16	1.59	14	2.12	1	0.57	1	0.60			
B*40:01	22	2.19	15	2.27	5	2.84	2	1.19			
B*40:02	65	6.47	41	6.21	7	3.98	17	10.12			0.0430
B*44:02	62	6.18	39	5.91	14	7.95	9	5.36			
B*48:01	32	3.19	26	3.94	1	0.57	5	2.98	0.0274		
B*49:01	13	1.29	9	1.36	1	0.57	3	1.79			
B*50:01	11	1.10	8	1.21	3	1.70	0	0.00			
B*51:01	65	6.47	36	5.45	21	11.93	8	4.76	0.0042		0.0279
B*52:01	16	1.59	8	1.21	4	2.27	4	2.38			
B*53:01	11	1.10	7	1.06	0	0.00	4	2.38			
**Others**	95	9.46	63	9.58	15	8.53	17	10.09			

AF: Allele Frequency. Only alleles with AF ≥ 1.0% are included in this table. *n*: Refers to the number of alleles. Only *p*-values < 0.05 are considered significant.

**Table 4:** *HLA-DRB1* allele frequency from Mexico: Tlalpan, Chihuahua, and Xalapa.

Allele	MM		Tlalpan		Chihuahua		Xalapa		*p*-Value		
									Tlalpan vs.		Chihuahua vs. Xalapa
	*n*	AF	*n*	AF	*n*	AF	*n*	AF	Chihuahua	Xalapa	
DRB1*01:01	35	3.49	23	3.48	9	5.11	3	1.79			
DRB1*01:02	29	2.89	22	3.33	6	3.41	1	0.60			
DRB1*03:01	49	4.88	36	5.45	11	6.25	2	1.19		0.0133	0.0205
DRB1*04:01	13	1.29	10	1.52	2	1.14	1	0.60			
DRB1*04:02	16	1.59	10	1.52	2	1.14	4	2.38			
DRB1*04:03	24	2.39	19	2.88	2	1.14	3	1.79			
DRB1*04:04	66	6.57	38	5.76	13	7.39	15	8.93			
DRB1*04:05	13	1.29	13	1.97	0	0.00	0	0.00			
DRB1*04:07	174	17.33	110	16.67	18	10.23	46	27.38	0.0466	0.0022	0.0001
DRB1*04:11	17	1.69	8	1.21	2	1.14	7	4.17		0.0251	
DRB1*07:01	74	7.37	53	8.03	15	8.52	6	3.57			
DRB1*08:02	100	9.96	73	11.06	13	7.39	14	8.33			
DRB1*10:01	13	1.29	9	1.36	3	1.70	1	0.60			
DRB1*11:01	36	3.59	27	4.09	7	3.98	2	1.19			
DRB1*11:04	13	1.29	6	0.91	3	1.70	4	2.38			
DRB1*13:01	33	3.29	20	3.03	7	3.98	6	3.57			
DRB1*13:02	10	1.00	7	1.06	1	0.57	2	1.19			
DRB1*13:03	10	1.00	6	0.91	4	2.27	0	0.00			
DRB1*14:01	15	1.49	3	0.45	9	5.11	3	1.79	<0.0001		
DRB1*14:02	49	4.88	29	4.39	9	5.11	11	6.55			
DRB1*14:06	51	5.08	37	5.61	11	6.25	3	1.79		0.0424	
DRB1*15:01	47	4.68	28	4.24	12	6.82	7	4.17			
DRB1*15:02	11	1.10	9	1.36	0	0.00	2	1.19			
DRB1*16:02	43	4.28	27	4.09	3	1.70	13	7.74			
**Others**	63	6.27	37	5.62	14	7.95	12	7.11			

AF: Allele Frequency. Only alleles with AF ≥ 1.0% are included in this table. *n*: Refers to the number of alleles. Only *p*-values < 0.05 are considered significant.

**Table 5:** *HLA-DQB1* allele frequency from Mexico: Tlalpan, Chihuahua, and Xalapa.

Allele	MM		Tlalpan		Chihuahua		Xalapa		*p*-Value		
									Tlalpan vs.		Chihuahua vs. Xalapa
	*n*	AF	*n*	AF	*n*	AF	*n*	AF	Chihuahua	Xalapa	
DQB1*02:01	78	7.77	59	8.94	14	7.95	5	2.98		0.0088	
DQB1*02:02	55	5.48	36	5.45	16	9.09	3	1.79		0.0426	0.0036
DQB1*03:01	222	22.11	145	21.97	43	24.43	34	20.24			
DQB1*03:02	316	31.47	202	30.61	40	22.73	74	44.05		0.0013	<0.0001
DQB1*04:02	118	11.75	82	12.42	14	7.95	22	13.10			
DQB1*05:01	91	9.06	62	9.39	21	11.93	8	4.76			0.0279
DQB1*06:01	29	2.89	19	2.88	4	2.27	6	3.57			
DQB1*06:02	36	3.59	22	3.33	10	5.68	4	2.38			
DQB1*06:03	31	3.09	16	2.42	10	5.68	5	2.98	0.0491		
**Others**	28	2.79	17	2.59	4	2.29	7	4.15			

AF: Allele Frequency. Only alleles with AF ≥ 1.0% are included in this table. *n*: Refers to the number of alleles. Only *p*-values < 0.05 are considered significant.

**Table 6:** *HLA-A-B-DRB1-DQB1* haplotypes from MM.

Haplotype	*n* (2*n* = 1004)	HF	∆′
A*02:01-B*35:01-DRB1*08:02-DQB1*04:02	17	1.69	0.1889
A*68:01-B*39:01-DRB1*04:07-DQB1*03:02	17	1.69	0.2429
A*02:01-B*35:01-DRB1*04:07-DQB1*03:02	12	1.20	0.0242
A*01:01-B*08:01-DRB1*03:01-DQB1*02:01	11	1.10	0.7747
A*68:01-B*39:01-DRB1*08:02-DQB1*04:02	11	1.10	0.1596

HF: Haplotype frequency. Only haplotypes with HF ≥ 1.0% are included in this table. **Δ′**: Standardized linkage disequilibrium. *n***:** Refers to the number of individuals analyzed.

## Supplementary Materials

Total alleles and haplotypes frequencies can be consulted in Supplementary materials https://www.mdpi.com/2075-4418/10/1/47/s1.
