# Supplementary material for: HLA Allele and Haplotype Frequencies in Three Urban Mexican Populations: Genetic Diversity for the Approach of Genomic Medicine
Source: Diagnostics (Basel). 2020 Jan 16;10(1):47. doi: 10.3390/diagnostics10010047 (PMC7168288; doi:10.3390/diagnostics10010047)
Supplement: Supplementary file 1 [file diagnostics-10-00047-s001.pdf]

Supplementary Table 1. *HLA-A* allele frequency from Mexico: Tlalpan, Chihuahua, and Xalapa

Allele	MM (n= 502)		Tlalpan (n=330)		Chihuahua (n=88)		Xalapa (n=84)	
	n	AF	n	AF	n	AF	n	AF
**A*01:01**	59	5.88	40	6.06	16	9.09	3	1.79
**A*02:01**	265	26.39	171	25.91	48	27.27	46	27.38
**A*02:02**	20	1.99	16	2.42	1	0.57	3	1.79
**A*03:01**	48	4.78	29	4.39	10	5.68	9	5.36
**A*11:01**	27	2.69	18	2.73	6	3.41	3	1.79
**A*11:04**	7	0.70	6	0.91	1	0.57	0	0.00
**A*23:01**	30	2.99	18	2.73	6	3.41	6	3.57
**A*24:02**	165	16.43	113	17.12	28	15.91	24	14.29
**A*25:01**	8	0.80	6	0.91	2	1.14	0	0.00
**A*26:01**	33	3.29	19	2.88	9	5.11	5	2.98
**A*29:01**	29	2.89	18	2.73	8	4.55	3	1.79
**A*30:01**	34	3.39	26	3.94	4	2.27	4	2.38
**A*31:01**	65	6.47	34	5.15	15	8.52	16	9.52
**A*31:03**	1	0.10	1	0.15	0	0.00	0	0.00
**A*32:01**	17	1.69	9	1.36	4	2.27	4	2.38
**A*33:01**	19	1.89	13	1.97	3	1.70	3	1.79
**A*34:04**	3	0.30	3	0.45	0	0.00	0	0.00
**A*36**	18	1.79	10	1.52	4	2.27	4	2.38
**A*66:01**	3	0.30	2	0.30	0	0.00	1	0.60
**A*68:01**	129	12.85	91	13.79	8	4.55	30	17.86
**A*68:02**	4	0.40	2	0.30	2	1.14	0	0.00
**A*68:03**	10	1.00	9	1.36	0	0.00	1	0.60
**A*68:05**	2	0.20	1	0.15	0	0.00	1	0.60
**A*69:01**	1	0.10	1	0.15	0	0.00	0	0.00
**A*74:01**	5	0.50	2	0.30	1	0.57	2	1.19

A*80:01	2	0.20	2	0.30	0	0.00	0	0.00

AF: Allele Frequency. Only alleles with. **n:** Refers to the number of the alleles

Supplementary Table 2. *HLA-B* allele frequency from Mexico: Tlalpan, Chihuahua, and Xalapa

Allele	MM (n= 502)		Tlalpan (n=330)		Chihuahua (n=88)		Xalapa (n=84)	
	**n**	**AF**	**n**	**AF**	**n**	**AF**	**n**	**AF**
**B*07:02**	47	4.68	23	3.48	16	9.09	8	4.76
**B*07:05**	1	0.10	1	0.15	0	0.00	0	0.00
**B*08:01**	30	2.99	20	3.03	9	5.11	1	0.60
**B*08:04**	1	0.10	0	0.00	1	0.57	0	0.00
**B*13:01**	12	1.20	9	1.36	2	1.14	1	0.60
**B*13:02**	1	0.10	1	0.15	0	0.00	0	0.00
**B*14:01**	24	2.39	12	1.82	9	5.11	3	1.79
**B*14:02**	25	2.49	22	3.33	3	1.70	0	0.00
**B*15:01**	54	5.38	36	5.45	7	3.98	11	6.55
**B*15:02**	10	1.00	10	1.52	0	0.00	0	0.00
**B*15:03**	8	0.80	4	0.61	1	0.57	3	1.79
**B*15:05**	4	0.40	4	0.61	0	0.00	0	0.00
**B*15:09**	1	0.10	0	0.00	1	0.57	0	0.00
**B*15:17**	1	0.10	0	0.00	1	0.57	0	0.00
**B*15:30**	1	0.10	0	0.00	0	0.00	1	0.60
**B*18:01**	25	2.49	15	2.27	7	3.98	3	1.79
**B*18:07**	2	0.20	2	0.30	0	0.00	0	0.00
**B*27**	9	0.90	6	0.91	2	1.14	1	0.60
**B*35:01**	161	16.04	109	16.52	19	10.80	33	19.64
**B*35:02**	43	4.28	28	4.24	5	2.84	10	5.95
**B*35:03**	1	0.10	0	0.00	0	0.00	1	0.60
**B*35:05**	1	0.10	1	0.15	0	0.00	0	0.00

**B*35:14**	3	0.30	2	0.30	1	0.57	0	0.00
**B*37:01**	7	0.70	6	0.91	0	0.00	1	0.60
**B*38:01**	11	1.10	3	0.45	6	3.41	2	1.19
**B*39:01**	131	13.05	86	13.03	20	11.36	25	14.88
**B*39:02**	23	2.29	21	3.18	1	0.57	1	0.60
**B*39:06**	16	1.59	14	2.12	1	0.57	1	0.60
**B*40:01**	22	2.19	15	2.27	5	2.84	2	1.19
**B*40:02**	65	6.47	41	6.21	7	3.98	17	10.12
**B*40:03**	1	0.10	0	0.00	0	0.00	1	0.60
**B*40:05**	5	0.50	1	0.15	1	0.57	3	1.79
**B*41:01**	4	0.40	4	0.61	0	0.00	0	0.00
**B*42:01**	2	0.20	1	0.15	1	0.57	0	0.00
**B*42:04**	1	0.10	1	0.15	0	0.00	0	0.00
**B*44:02**	62	6.18	39	5.91	14	7.95	9	5.36
**B*45:01**	7	0.70	5	0.76	2	1.14	0	0.00
**B*45:02**	3	0.30	3	0.45	0	0.00	0	0.00
**B*46:01**	1	0.10	1	0.15	0	0.00	0	0.00
**B*48:01**	32	3.19	26	3.94	1	0.57	5	2.98
**B*49:01**	13	1.29	9	1.36	1	0.57	3	1.79
**B*49:02**	3	0.30	3	0.45	0	0.00	0	0.00
**B*50:01**	11	1.10	8	1.21	3	1.70	0	0.00
**B*51:01**	65	6.47	36	5.45	21	11.93	8	4.76
**B*51:02**	1	0.10	0	0.00	0	0.00	1	0.60
**B*52:01**	16	1.59	8	1.21	4	2.27	4	2.38
**B*52:02**	2	0.20	1	0.15	1	0.57	0	0.00
**B*53:01**	11	1.10	7	1.06	0	0.00	4	2.38
**B*54:01**	1	0.10	1	0.15	0	0.00	0	0.00
**B*55:01**	6	0.60	6	0.91	0	0.00	0	0.00
**B*55:02**	1	0.10	0	0.00	1	0.57	0	0.00
**B*56:01**	1	0.10	1	0.15	0	0.00	0	0.00

**B*57:01**	6	0.60	5	0.76	0	0.00	1	0.60
**B*57:02**	2	0.20	2	0.30	0	0.00	0	0.00
**B*58:01**	6	0.60	1	0.15	2	1.14	3	1.79
**B*78:01**	1	0.10	0	0.00	0	0.00	1	0.60

AF: Allele Frequency. Only alleles with. **n:** Refers to the number of alleles.

Supplementary Table 3. *HLA-DRB1* allele frequency from Mexico: Tlalpan, Chihuahua, and Xalapa

**Allele**	**MM (n= 502)**		**Tlalpan (n=330)**		**Chihuahua (n=88)**		**Xalapa (n=84)**	
	**n**	**AF**	**n**	**AF**	**n**	**AF**	**n**	**AF**
**DRB1*01:01**	35	3.49	23	3.48	9	5.11	3	1.79
**DRB1*01:02**	29	2.89	22	3.33	6	3.41	1	0.60
**DRB1*01:03**	4	0.40	2	0.30	0	0.00	2	1.19
**DRB1*03:01**	49	4.88	36	5.45	11	6.25	2	1.19
**DRB1*03:02**	2	0.20	1	0.15	0	0.00	1	0.60
**DRB1*03:03**	3	0.30	3	0.45	0	0.00	0	0.00
**DRB1*04:01**	13	1.29	10	1.52	2	1.14	1	0.60
**DRB1*04:02**	16	1.59	10	1.52	2	1.14	4	2.38
**DRB1*04:03**	24	2.39	19	2.88	2	1.14	3	1.79
**DRB1*04:04**	66	6.57	38	5.76	13	7.39	15	8.93
**DRB1*04:05**	13	1.29	13	1.97	0	0.00	0	0.00
**DRB1*04:07**	174	17.33	110	16.67	18	10.23	46	27.38
**DRB1*04:08**	1	0.10	0	0.00	0	0.00	1	0.60
**DRB1*04:10**	2	0.20	2	0.30	0	0.00	0	0.00
**DRB1*04:11**	17	1.69	8	1.21	2	1.14	7	4.17
**DRB1*04:13**	2	0.20	0	0.00	2	1.14	0	0.00
**DRB1*07:01**	74	7.37	53	8.03	15	8.52	6	3.57
**DRB1*07:05**	1	0.10	1	0.15	0	0.00	0	0.00

**DRB1*07:08**	1	0.10	0	0.00	0	0.00	1	0.60
**DRB1*08:01**	7	0.70	4	0.61	1	0.57	2	1.19
**DRB1*08:02**	100	9.96	73	11.06	13	7.39	14	8.33
**DRB1*08:04**	2	0.20	1	0.15	1	0.57	0	0.00
**DRB1*08:10**	2	0.20	2	0.30	0	0.00	0	0.00
**DRB1*09:01**	6	0.60	5	0.76	1	0.57	0	0.00
**DRB1*10:01**	13	1.29	9	1.36	3	1.70	1	0.60
**DRB1*11:01**	36	3.59	27	4.09	7	3.98	2	1.19
**DRB1*11:02**	5	0.50	3	0.45	1	0.57	1	0.60
**DRB1*11:04**	13	1.29	6	0.91	3	1.70	4	2.38
**DRB1*12:01**	6	0.60	2	0.30	3	1.70	1	0.60
**DRB1*13:01**	33	3.29	20	3.03	7	3.98	6	3.57
**DRB1*13:02**	10	1.00	7	1.06	1	0.57	2	1.19
**DRB1*13:03**	10	1.00	6	0.91	4	2.27	0	0.00
**DRB1*13:04**	1	0.10	1	0.15	0	0.00	0	0.00
**DRB1*13:05**	2	0.20	1	0.15	1	0.57	0	0.00
**DRB1*13:10**	3	0.30	0	0.00	2	1.14	1	0.60
**DRB1*13:16**	1	0.10	0	0.00	1	0.57	0	0.00
**DRB1*14:01**	15	1.49	3	0.45	9	5.11	3	1.79
**DRB1*14:02**	49	4.88	29	4.39	9	5.11	11	6.55
**DRB1*14:04**	2	0.20	2	0.30	0	0.00	0	0.00
**DRB1*14:06**	51	5.08	37	5.61	11	6.25	3	1.79
**DRB1*15:01**	47	4.68	28	4.24	12	6.82	7	4.17
**DRB1*15:02**	11	1.10	9	1.36	0	0.00	2	1.19
**DRB1*15:03**	3	0.30	1	0.15	0	0.00	2	1.19
**DRB1*15:06**	1	0.10	0	0.00	1	0.57	0	0.00
**DRB1*16:01**	6	0.60	6	0.91	0	0.00	0	0.00
**DRB1*16:02**	43	4.28	27	4.09	3	1.70	13	7.74

AF: Allele Frequency. Only alleles with. **n:** Refers to the number of alleles.

Supplementary Table 4. *HLA-DQB1* allele frequency from Mexico: Tlalpan, Chihuahua, and Xalapa

Allele	MM (n= 502)		Tlalpan (n=330)		Chihuahua (n=88)		Xalapa (n=84)	
	**n**	**AF**	**n**	**AF**	**n**	**AF**	**n**	**AF**
**DQB1*02:01**	78	7.77	59	8.94	14	7.95	5	2.98
**DQB1*02:02**	55	5.48	36	5.45	16	9.09	3	1.79
**DQB1*03:01**	222	22.11	145	21.97	43	24.43	34	20.24
**DQB1*03:02**	316	31.47	202	30.61	40	22.73	74	44.05
**DQB1*03:03**	6	0.60	4	0.61	0	0.00	2	1.19
**DQB1*03:04**	6	0.60	4	0.61	0	0.00	2	1.19
**DQB1*04:02**	118	11.75	82	12.42	14	7.95	22	13.10
**DQB1*04:03**	1	0.10	0	0.00	1	0.57	0	0.00
**DQB1*05:01**	91	9.06	62	9.39	21	11.93	8	4.76
**DQB1*05:02**	3	0.30	2	0.30	0	0.00	1	0.60
**DQB1*05:03**	3	0.30	0	0.00	3	1.70	0	0.00
**DQB1*06:01**	29	2.89	19	2.88	4	2.27	6	3.57
**DQB1*06:02**	36	3.59	22	3.33	10	5.68	4	2.38
**DQB1*06:03**	31	3.09	16	2.42	10	5.68	5	2.98
**DQB1*06:04**	6	0.60	5	0.76	0	0.00	1	0.60
**DQB1*06:09**	3	0.30	2	0.30	0	0.00	1	0.60

AF: Allele Frequency. Only alleles with. **n:** Refers to the number of alleles.

Supplementary Table 5: *HLA-A-B-DRB1-DQB1* haplotypes of the three regions from Mexico

Tlalpan	n=(2n=660)	HF (%)	Δ'
**A*02:01-B*35:01-DRB1*08:02-DQB1*04:02**	13	1.97	0.2209
**A*68:01-B*39:01-DRB1*04:07-DQB1*03:02**	13	1.97	0.2389
**A*68:01-B*39:01-DRB1*08:02-DQB1*04:02**	10	1.52	0.1935
**A*01:01-B*08:01-DRB1*03:01-DQB1*02:01**	7	1.06	0.8678
**A*02:01-B*35:01-DRB1*04:07-DQB1*03:02**	7	1.06	0.0026
**Chihuahua**	**n=(2n=176)**	**HF (%)**	**Δ'**
**A*01:01-B*08:01-DRB1*03:01-DQB1*02:01**	4	2.27	0.6444
**A*24:02-B*39:01-DRB1*04:07-DQB1*03:02**	3	1.70	0.5572
**A*01:01-B*14:01-DRB1*01:02-DQB1*05:01**	2	1.14	0.3788
**A*02:01-B*51:01-DRB1*14:06-DQB1*03:01**	2	1.14	0.2472
**A*03:01-B*07:02-DRB1*15:01-DQB1*06:02**	2	1.14	0.4731
**A*02:01-B*51:01-DRB1*01:01-DQB1*05:01**	2	1.14	0.2472
**A*29:01-B*44:02-DRB1*07:01-DQB1*02:02**	2	1.14	0.4601
**Xalapa**	**n=(2n=168)**	**H.F.**	**Δ'**
**A*24:02-B*35:01-DRB1*04:07-DQB1*03:02**	5	2.98	0.6098
**A*02:01-B*35:01-DRB1*04:07-DQB1*03:02**	4	2.38	0.0544
**A*02:01-B*35:01-DRB1*08:02-DQB1*04:02**	4	2.38	0.2448
**A*68:01-B*39:01-DRB1*04:07-DQB1*03:02**	3	1.79	0.2195
**A*02:01-B*35:02-DRB1*04:07-DQB1*03:02**	3	1.79	0.6585
**A*31:01-B*39:01-DRB1*08:02-DQB1*04:02**	2	1.19	0.4545
**A*68:01-B*40:02-DRB1*04:07-DQB1*03:02**	2	1.19	0.5447
**A*24:02-B*35:01-DRB1*14:02-DQB1*03:01**	2	1.19	0.2453
**A*68:01-B*35:01-DRB1*04:04-DQB1*03:02**	2	1.19	0.1818
**A*02:01-B*40:02-DRB1*04:07-DQB1*03:02**	2	1.19	0.3171

HF: Haplotype frequency. Only haplotypes with HF ≥ 1.0% are included in this table. **Δ′**: Standardized linkage disequilibrium. **n:** Refers to the number of individuals analyzed.
